# Orthopaedic surgeons' attitude toward physical activity for people after total hip or knee replacement: Northern vs Southern European country

**DOI:** 10.1186/s12891-024-07488-w

**Published:** 2024-05-11

**Authors:** Raffaele Zinno, Inge van den Akker-Scheek, Erika Pinelli, PAIR study group, Alessandro Mazzotta, Alina Iliescu, Andrea Fabio Manunta, Andreea Marin, Ani Dimitrova, Ann-Katrin Stensdotter, Cristiano Paggetti, Dante Dallari, Elena Tamburini, Francesco Benvenuti, Francesco Pegreffi, Giuseppe Barone, Havard Østerås, Ileana Ciobanu, Ivo Dimitrov, Jorunn Laegdheim Helbostad, Lora Yoncheva, Maria Scoppolini Massini, Matei Teodorescu, Maya Tsvetanova, Mihai Berteanu, Monica Unsgaard-Tøndel, Natalya Shalamanova, Nicolay Todorov, Odd Magne Hals, Rumyana Shalamanova, Simona Geli, Umberto Cardinale, Yvet Mooiweer, Laura Bragonzoni, Martin Stevens

**Affiliations:** 1 Department for Life Quality Studies, University of Bologna, Via Di Barbiano 1/10, 47921 Rimini, Italy; 2 Department of Orthopaedics, University of Groningen, University Medical Center Groningen, Groningen, The Netherlands

**Keywords:** Physical activity, Sports, Orthopaedics, Italy, The Netherlands, Total knee replacement, Total hip replacement

## Abstract

**Background:**

Regular physical activity (PA) is a key factor of lifestyle behavior enhancing general health and fitness, especially in people after total hip or knee replacement (THR and TKR). Orthopaedic surgeons can play a primary role in advocating the benefits of an active lifestyle. Aim of the study was 1) to assess the attitude of orthopaedic surgeons towards PA for people after THR/TKR and 2) to compare the attitude between a Northern European (the Netherlands) and a Southern European (Italy) country and analyze which factors influence the attitude towards PA.

**Methods:**

A cross-cultural study. An (online) survey was distributed among orthopaedic surgeons in Italy and the Netherlands. Chi-square and Mann–Whitney tests were used to compare surgeons’ and clinics’ characteristics, and questionnaires’ scores, respectively. A linear regression analysis was conducted to assess which surgeon characteristics influence attitude towards PA.

**Results:**

A cohort of 159 surgeons (103 Italians and 56 Dutch) was analyzed. The median score of overall orthopaedic surgeons’ attitude towards PA was positive (57 out of 72). Dutch surgeons showed a more positive attitude compared to Italian surgeons (*p* < 0.01). Main difference was found in the “Physical activity concern” factor, where Italian surgeons showed more concern about the negative effects of PA on the survival of the prosthesis. The regression analyses showed that “Country” and “Type of clinic” were associated with the surgeons’ attitude.

**Conclusions:**

Overall, the orthopaedic surgeons’ attitude towards PA for people with THR and TKR was positive. However, Dutch surgeons seem to be more positive compared to the Italian. The country of residence was the item that most influenced attitude. Further investigations are needed to untangle specific factors, such as cultural, socioeconomic, or contextual differences within the variable “country” that may influence orthopaedic surgeons’ attitudes towards PA.

**Supplementary Information:**

The online version contains supplementary material available at 10.1186/s12891-024-07488-w.

## Background

Osteoarthritis (OA) is the most prevalent form of arthritis and mainly affects hip and knee joints [1]. More than 50% of people with OA suffer from medium or severe pain, with reduced performance in the activities of daily living, social participation and lower levels of quality of life [2]. The preferred treatment for end-stage OA is total hip and knee replacement (THR and TKR) [3, 4]. Indeed, these surgical procedures are among the most cost-effective treatments available [4, 5]. However, the increased number of people requiring surgery results in high socio-economic costs both for the procedure itself and the subsequent rehabilitation treatment [6].

The Organization for Economic Co-operation and Development (OECD) reported 193.9 THR and 137.0 TKR cases in 2019 per 100,000 population in Italy, while the Netherlands reported 222.3 THR and 171.4 TKR cases in 2019 [7]. The incidence of OA is increasing mainly due to the aging society, the obesity epidemic, and a physically inactive lifestyle [1, 8, 9]. Given the increase of such risk factors, the number of surgical procedures related to OA will proportionally increase, as well as the direct and indirect economic burden [6, 10, 11].

Regular physical activity (PA) is recognized as a key factor of lifestyle behavior enhancing general health and fitness [12]. Indeed, PA can help to prevent overweight, obesity, and hypertension which represent modifiable risk factors for several chronic diseases, including cardiovascular diseases, diabetes, and certain cancers. Specific for patients after THR/TKR, a physically active lifestyle can be beneficial in terms of increased mineral bone density, improved prosthetic fixation, reduced risk of prosthetic loosening and a lower fall risk [12, 13]. Therefore, performing regular PA is even more important for people with THR and TKR. Finally PA has also an impact on fitness, which is associated with functional autonomy and, consequently, with longer independence in older adults [12].

Currently, there are no international guidelines concerning PA behavior for people after THR and TKR. However, there are PA guidelines for the general population, i.e., the WHO recommends all adults perform at least 150 to 300 min of moderate aerobic activity per week (or the equivalent vigorous activity). Additionally, it is recommended that adults perform muscle-strengthening activities at least twice a week, and older adults should also include balance exercises [14]. Since people after total joint replacement may be considered to be “healthy” again, such guidelines seem also applicable to people with THR and TKR [12, 15]. In addition, orthopaedic surgeons showed consensus in allowing people after THR and TKR to return to low-impact sports activities such as walking, swimming, and biking on level surfaces [4, 16, 17]. On the other hand, surgeons do not recommend contact sports, most of the ball sports, and martial arts [4, 12].

Orthopaedic surgeons can have a primary role in discussing and making people aware of the impact of an active lifestyle for the sake of general health, fitness and the longevity of the prosthesis itself [4, 18, 19]. However, until now health care professionals may not counsel patients enough about PA or they provide general advice only [4, 20]. Consequently health care professionals do need to improve the quality and quantity of exercise counseling [19]. However, this counseling seems to be influenced by their own attitude towards PA [21].

This attitude can be influenced by cultural characteristics, i.e. the level of PA of the general population in a particular country. Among European countries there are significant differences in levels of sport, exercise, and PA participation [22]. It appears that Southern European countries report lower levels of sports, exercise, and physical activity participation than their Northern European counterparts [22]. In particular, in Italy, the proportion of adults who reported engaging in sports, exercise, or other physical activities at least once a week in 2022 was lower than the European average, 34 versus 38%. In contrast, the Netherlands reported a higher level (60%) of sports, exercise, or other physical activities than European countries [22]. It can be hypothesized that these differences are of influence on orthopaedic surgeons’ attitude towards PA and consequently their counseling of people after THR/TKR [23, 24].

In light of this, the primary aim of this study was to assess the attitude of orthopaedic surgeons towards PA for people after THR/TKR. Secondly to compare the attitude between surgeons from a Northern European (the Netherlands) and a Southern European (Italy) country and analyze what are the factors that influence the attitude towards PA.

## Methods

A cross-cultural study that examines the attitude of Italian and Dutch orthopaedic surgeons towards PA for people after THR and TKR was performed. The study was conducted according to the Strengthening the Reporting of Observational Studies in Epidemiology (STROBE) for cross-sectional studies (Supplementary materials—Tab. S1).

The study is part of a wider European project (“Physical ActIvity after total hip and knee Replacement”, PAIR project) funded within the Erasmus Plus Sport program (Grant Agreement 613,008-EPP-1–2019-1-IT-SPO-SCP). The Physical Activity after knee or hip Replacement (PAIR) group, which collaborated on the design and data collection of the present study, is composed by University of Bologna located in Bologna (Italy), Rizzoli Orthopaedic Institute (IOR) located in Bologna (Italy), Medea located in Florence (Italy), Carol Davila University of Medicine and Pharmacy located in Bucharest (Romania), Norwegian University of Science and Technology (NTNU), University Medical Center Groningen (UMCG) located in Groningen (the Netherlands), and Know and Can association located in Sofia (Bulgaria). The present study was focused on data that concerns the attitude towards PA of Italian and Dutch orthopaedic surgeons.

Participants signed an informed consent, and the study was executed in accordance with the Helsinki Declaration. Partners signed a data processor agreement allowing NTNU to process data on the behalf of the data controller. A general ethical approval for the survey was granted for NTNU (REK 244244 / 25.08.2021). Moreover, the study was approved in Italy by the Local Ethics Committee (Comitato Etico Indipendente di Area Vasta Emilia Centro, CE-AVEC) of the Emilia-Romagna Region, Italy (reference number AVEC: 1005/2020/Sper/IOR) and registered in ClinicalTrial.Gov (NCT04761367), and by the Medical Ethical Committee of UMCG (ref nr 2020/530) in the Netherlands.

### Questionnaire

For the purpose of this study, a questionnaire assessing the orthopaedic surgeons’ attitude toward PA for people after THR and TKR was developed (Supplementary materials—Tab. S2, S3, S4, and S5). The questionnaire was composed of 37 items and is divided into 4 sections: 1) background, 2) personal information, 3) health service, and 4) attitude towards PA. Sections 1 to 3 were necessary for analyzing and providing the background and context in which the surgeons work; they comprised 4, 6, and 9 items respectively. Section 4 was composed of 18 items which provide a description of the attitude toward PA for people after THR and TKR and responses can be scored on a 4-point Likert scale (1 to 4 scores, from totally disagree to totally agree). The minimum total score was 18, while the maximum was 72.

A score ranging from 54 to 72, corresponding to over 75% of the maximum score, was considered as a positive attitude; a score between 36 and 53, corresponding to 50%-75% of the maximum score, was considered a neutral attitude; and a score below 36 was considered a negative attitude.

The questionnaire was developed by NTNU. Then it was translated into English and shared with the Dutch and Italian PAIR partners. Cross-cultural linguistic adaptation, translation, and back-translation processes were performed in each country [25]. The translated and back-translated (in English) versions were reviewed by an internal committee of the PAIR consortium. The final version was then pre-tested in a small sample of the target population. Finally, after the approval of the partners, the questionnaires were administered to the target population in their native languages.

### Administration of the questionnaire

The final version of the questionnaire was completed in March 2021. Different approaches for the administration of the questionnaire were used. In Italy, the snowball sampling methodology [26] for recruitment was used. Moreover, the administration of the questionnaire to the orthopaedic surgeons was performed through two modalities: 1) Web-based modality, where the link for the online questionnaire (i.e., SurveyMonkey) was sent to orthopaedic surgeons who expressed their will to participate. The digital platform used for collecting the responses was approved by the General Data Protection Regulation. 2) On paper-based modality, where the questionnaire was printed and provided to the surgeons manually. Returned questionnaires were subsequently copied or scanned and delivered by mail to NTNU. The responses were manually entered into the GDPR-approved WebCRF database at NTNU where each partner country was given an account. In the Netherlands, the surgeons were invited by means of the weekly newsletter of the Dutch Orthopaedic Association and through the personal network of the involved researchers. Concerning the questionnaires administration, the web-based modality through Research Electronic Data Capture (REDCap) was used. Using REDCap was approved by the General Data Protection Regulation of UMCG.

The responders were anonymous since there were no person-identifiable items in the questionnaire and all the data have been analyzed in aggregated form.

### Statistics

Demographic characteristics were analyzed with descriptive statistics using mean and SD or frequency and percentage as appropriate. The Chi-square test was used to compare the surgeons’ and clinics’ characteristics between countries. Exploratory factor analysis (EFA) was performed to gain insight into the latent factor structure behind the items of section 4 (“Attitude towards physical activity”) of the questionnaire, ultimately facilitating the identification of relevant factors that influence the attitude of orthopaedic surgeons towards PA for individuals with a total knee or hip prosthesis. The factor solution was rotated using varimax rotation to enhance the interpretability. Prior to factor extraction, the adequacy of the data for factor analysis was assessed using Kaiser–Meyer–Olkin index and Bartlett's test of sphericity. The eigenvalue criterion greater than 1 was employed to determine the optimal number of factors to be retained.

### Attitude towards physical activity

The median of the total score, obtained by summing each item of the “Attitude towards physical activity” section, was compared between countries by the Mann Whitney test.

### Comparison between a Northern and Southern European country

As for the total score, the median score of each identified factor, obtained by summing all items related to that factor, was compared between countries by the Mann Whitney test.

### Factors influencing attitude towards physical activity

To assess the factors influencing the attitude of orthopaedic surgeons towards PA, a multiple linear regression analysis was conducted on the total score and on those factors that exhibited a significant difference between countries. The dependent variable was the attitude of orthopaedic surgeons towards PA. The independent variables were surgeons’ characteristics (residence area, type of clinic, gender, age, educational level, working experiences, sport participation, and country). Adjusted R-squared was used as a measure of model fit, representing the proportion of variance in the dependent variable explained by the independent variables. For all the statistical analysis, the version 28.0.0.0 (190) of IBM SPSS (IBM Corp., Armonk, NY, USA) software was used. Missing data were managed by excluding cases analysis by analysis. A *P*-value < 0.05 was considered to indicate statistical significance.

## Results

A cohort of 159 surgeons (103 Italian and 56 Dutch) completed and returned the questionnaire. The analysis of the sample characteristics showed that the majority of participants worked in urban areas, specifically in orthopaedic clinics and hospital policlinics. The age distribution revealed that the largest group fell within the age range of 31 to 40 years and that most of the participants were male. Regarding educational qualifications, 37.7% of the participants had obtained a PhD degree. In terms of work experience, 61.2% had more than 10 years of experience in health service and 56.3% had more than 10 years of experience with THR and TKR patient groups. Concerning sport and PA participation, 56.6% of the surgeons were involved in moderate or regular PA.

The comparison between Italian and Dutch surgeons concerning the “Background” and “personal information” sections showed significant differences related to working area, type of clinic where surgeons work, educational level, and sport/physical activity participation. In particular, Italian surgeons were more likely working in Urban areas than Dutch surgeons. Moreover, Dutch surgeons worked more frequently in Hospital, while Italian ones worked more frequently in specialized orthopaedic clinics.

Concerning the educational level, Dutch surgeons showed a higher level of education, which in our study means the possession of a PhD., a higher level of PA, and sport participation compared to the Italian surgeons. There were no statistically significant differences in gender, age, and work experience between countries (Table 1).

**Table 1:** Sample characteristics

Questions	Answers	Total n° (%)	Italy n° (%)	The Netherlands n° (%)	Pearson Chi^2^	*P* value
Sample		159	104	55		
Area of clinic	Rural	18 (11.9)	2 (2.1)	16 (29.6)	31.925	** < 0.001**
	Suburban	13 (8.6)	5 (5.2)	8 (14.8)		
	Urban	120 (79.5)	90 (92.8)	30 (55.6)		
Type of clinic	Orthopaedic clinic	74 (46.5)	55 (52.9)	19 (34.5)	14.047	** < 0.001**
	Hospital policlinic	71 (44.8)	36 (34.6)	35 (63.6)		
	Community health clinic	14 (8.8)	13 (12.5)	1 (1.8)		
Gender	Male	131 (86.2)	86 (87.8)	45 (83.3)	0.572	0.450
	Female	21 (13.8)	12 (12.2)	9 (16.7)		
Age (in years)	< 30	8 (5.2)	5 (5.1)	3 (5.6)	4.228	0.376
	31–40	65 (42.5)	43 (43.4)	22 (40.7)		
	41–50	37 (24.2)	22 (22.2)	15 (27.8)		
	51–60	32 (20.9)	19 (19.2)	13 (24.1)		
	61–70	11 (7.2)	10 (10.1)	1 (1.9)		
Educational level	Master’s degree	96 (62.3)	73 (73.0)	23 (42.6)	13.809	** < 0.001**
	PhD degree	58 (37.7)	27 (27.0)	31 (57.4)		
Work experience – Health service (in years)	< 1	1 (0.7)	1 (1.0)	0 (0.0)	2.843	0.584
	1–5	23 (15.1)	18 (18.2)	5 (9.4)		
	6–10	35 (23.0)	21 (21.2)	14 (26.4)		
	11–20	48 (31.6)	30 (30.3)	18 (34.0)		
	> 20	45 (29.6)	29 (30.3)	16 (30.2)		
Work experience – Patient group (in years)	< 1	4 (2.6)	3 (3.1)	1 (1.9)	3.256	0.516
	1–5	34 (22.5)	24 (24.7)	10 (18.5)		
	6–10	28 (18.5)	19 (19.6)	9 (16.7)		
	11–20	48 (31.8)	26 (26.8)	22 (40.7)		
	> 20	37 (24.5)	25 (25.8)	12 (22.2)		
Sport/Physical activity participation	None	8 (5.2)	8 (8.0)	0 (0.0)	11.081	**0.011**
	Leisure/Irregular	48 (31.2)	37 (37.0)	11 (20.4)		
	Moderate/Regular	87 (56.6)	48 (48.0)	39 (72.2)		
	High/Competitive	11 (7.1)	7 (7.0)	4 (7.4)		

Statistically significant *p* value in bold

Concerning the “health service” section, the main differences between both countries were associated with the duration of exercise classes offered by the clinic, the presence and duration of pre-operative and post-operative exercise, the tool employed for information dissemination, and the provision of advice regarding smoking. In particular, Dutch clinics give more frequently advise on quitting smoking, and all the information for patients is provided through written and oral formats. Moreover, Dutch clinics offered more frequent pre-operative exercise programs and of longer duration than those offered by Italian clinics. On the other hand, Italian clinics reported offering a higher frequency of daily PA classes and longer post-operative exercise programs for people with total hip and knee prosthesis (Table 2).

**Table 2:** Clinics characteristics

Questions	Answers		Total n°(%)	Italy n°(%)	The Netherlands n°(%)	Pearson Chi^2^	*P* value
Sample			159	104	55		
The clinic offers exercise classes for this patient group	None		102 (74.5)	63 (73.3)	39 (76.5)	8.743	** < 0.05**
	Daily		20 (14.6)	17 (19.8)	3 (5.9)		
	Weekly		3 (2.2)	2 (2.3)	1 (2.0)		
	Occasional		12 (8.8)	4 (4.7)	8 (15.7)		
The clinic offers a pre-operative exercise program	None		104 (75.9)	72 (83.7)	32 (62.7)	7.813	** < 0.05**
	2 < 3 times introductory		25 (18.2)	11 (12.8)	14 (27.5)		
	3 Weekly 1–2 months		8 (5.8)	3 (3.5)	5 (9.8)		
The clinic offers a post-operative exercise program	None		47 (34.3)	17 (19.8)	30 (58.8)	24.488	** < 0.001**
	2 < 3 times introductory		38 (27.7)	27 (31.4)	11 (21.6)		
	3 Weekly 1–2 months		45 (32.8)	38 (44.2)	7 (13.7)		
	4 Weekly > 2 months		7 (5.1)	4 (4.7)	3 (5.9)		
Advice and supervision are individually personalized	No		42 (30.7)	29 (33.7)	13 (25.5)	1.020	0.312
	Yes		95 (69.3)	57 (66.3)	38 (74.5)		
The information is given	None		2 (1.5)	2 (2.3)	0 (0.0)	25.125	** < 0.001**
	Orally		26 (19.0)	23 (26.7)	3 (5.9)		
	Written		15 (10.9)	15 (17.4)	0 (0.0)		
	Orally and written		94 (68.6)	46 (53.5)	48 (94.1)		
I give physical activity advice	Not my job		1 (0.7)	1 (1.2)	0 (0.0)	3.251	0.354
	Never		1 (0.7)	1 (1.2)	0 (0.0)		
	Sometimes		41 (30.1)	22 (25.6)	19 (38.0)		
	Always		93 (68.4)	62 (72.1)	31 (62.0)		
I give smoke secession advice	Not my job		8 (5.8)	8 (9.3)	0 (0.0)	18.793	** < 0.001**
	Never		29 (21.2)	26 (30.2)	3 (5.9)		
	Sometimes		60 (43.8)	32(37.2)	28 (54.9)		
	Always		40 (29.2)	20 (23.3)	20 (39.2)		
I give weight reduction advice	Not my job		5 (3.6)	5 (5.8)	0 (0.0)	7.514	0.057
	Never		3 (2.2)	2 (2.3)	1 (2.0)		
	Sometimes		60 (43.8)	31 (36.0)	29 (56.9)		
	Always		69 (50.4)	48 (55.8)	21 (41.2)		
The clinic gives information about importance of physical activity by:	None	No	146 (91.8)	94 (90.4)	52 (94.5)	0.830	0.362
		Yes	13 (8.2)	10 (9.6)	3 (5.5)		
	Physician	No	63 (39.6)	41 (39.4)	22 (40.0)	0.005	0.944
		Yes	96 (60.4)	63 (60.6)	33 (60.0)		
	Secretary	No	155 (97.5)	104 (100)	51 (97.5)	7.759	** < 0.01**
		Yes	4 (2.5)	0 (0.0)	4 (2.5)		
	Nurse	No	133 (83.6)	101 (97.1)	32 (58.2)	39.867	** < 0.001**
		Yes	26 (16.4)	3 (2.9)	23 (41.8)		
	Physio therapist	No	72 (45.3)	55 (52.9)	17 (30.9)	7.012	** < 0.01**
		Yes	87 (54.7)	49 (47.1)	38 (69.1)		
	Exercise trainer (non-medical)	No	157 (98.7)	103 (99.0)	54 (98.2)	0.213	0.645
		Yes	2 (1.3)	1 (1.0)	1 (1.8)		
	Occupational therapist	No	158 (99.4)	103 (99.0)	55 (100)	0.532	0.466
		Yes	1 (0.6)	1 (1.0)	0 (0.0)		

Statistically significant *p* value in bold

### Attitude towards physical activity

The EFA was performed on the items within the “Attitude towards physical activity” section, identifying five factors which were labelled as: (1) Importance of PA, (2) Physical activity participation, (3) Physical activity concern, (4) Physical functioning, and (5) Knowledge (Table 3). Together the five factors explained 64.4% of the variance. The description of the factor loadings is provided in the supplementary material (Supplementary materials, Tab. S6-7).

**Table 3:** Factors descriptions

	N	Questions	Answers	Total n° (%)	Italy n° (%)	The Netherlands n° (%)	Pearson Chi^2^	*P* value
**Factor 1—Importance of PA**	Q20	Physical activity is important for general health	Strongly disagree	0 (0.0)	0 (0.0)	0 (0.0)	0.004	0.948
			Disagree	0 (0.0)	0 (0.0)	0 (0.0)		
			Agree	15 (11.5)	9 (11.4)	6 (11.8)		
			Totally agree	115 (88.5)	70 (88.6)	45 (88.2)		
	Q21	Physical activity is important for physical function	Strongly disagree	0 (0.0)	0 (0.0)	0 (0.0)	0.001	0.974
			Disagree	0 (0.0)	0 (0.0)	0 (0.0)		
			Agree	18 (13.8)	11 (13.9)	7 (13.7)		
			Totally agree	112 (86.2)	68 (86.1)	44 (86.3)		
	Q22	Physical activity is important for quality of life	Strongly disagree	0 (0.0)	0 (0.0)	0 (0.0)	1.738	0.419
			Disagree	1 (0.8)	0 (0.0)	1 (0.8)		
			Agree	21 (16.2)	12 (15.2)	9 (17.6)		
			Totally agree	108 (83.1)	67 (84.8)	41 (80.4)		
	Q23	Physical activity is for everyone	Strongly disagree	0 (0.0)	0 (0.0)	0 (0.0)	2.837	0.242
			Disagree	9 (7.1)	7 (9.1)	2 (4.0)		
			Agree	45 (35.4)	30 (39.0)	15 (30.0)		
			Totally agree	73 (57.5)	40 (51.9)	33 (66.0)		
**Factor 2—Physical activity participation**	Q35	Exercises for muscle strength is important for function for this patient group	Strongly disagree	0 (0.0)	0 (0.0)	0 (0.0)	2.041	0.360
			Disagree	3 (2.3)	1 (1.3)	2 (3.9)		
			Agree	77 (60.2)	44 (57.1)	77 (60.2)		
			Totally agree	48 (37.5)	32 (41.6)	48 (37.5)		
	Q36	Physical activity is important to enable participation (social, work, leisure)	Strongly disagree	0 (0.0)	0 (0.0)	0 (0.0)	1.838	0.399
			Disagree	1 (0.8)	1 (1.3)	0 (0.0)		
			Agree	83 (65.9)	47 (61.8)	36 (72.0)		
			Totally agree	42 (33.3)	28 (36.8)	14 (28.8)		
	Q37	Physical activity is important for coping with having prosthesis	Strongly disagree	0 (0.0)	0 (0.0)	0 (0.0)	7.489	**0.024**
			Disagree	10 (7.8)	9 (11.5)	1 (2.0)		
			Agree	87 (67.4)	46 (59.0)	41 (80.4)		
			Totally agree	32 (24.8)	23 (29.5)	9 (17.6)		
**Factor 3—Physical activity concern**	Q24	The prosthesis alone restores full physical function	Strongly disagree	14 (10.8)	13 (16.5)	1 (2.0)	42.605	** < 0.001**
			Disagree	47 (36.2)	41 (51.9)	6 (11.8)		
			Agree	49 (37.7)	22 (27.8)	27 (52.9)		
			Totally agree	20 (15.4)	3 (3.8)	17 (33.3)		
	Q25	Physical activity is not necessary	Strongly disagree	68 (52.7)	62 (79.5)	6 (11.8)	94.930	** < 0.001**
			Disagree	17 (13.2)	15 (19.2)	2 (3.9)		
			Agree	11 (8.5)	1 (1.3)	10 (19.6)		
			Totally agree	33 (25.6)	0 (0.0)	33 (64.7)		
	Q26	Vigorous physical activity may damage the prosthesis	Strongly disagree	9 (7.0)	3 (3.8)	6 (11.8)	5.337	0.149
			Disagree	43 (33.3)	23 (29.5)	20 (39.2)		
			Agree	66 (51.2)	45 (57.7)	21 (41.2)		
			Totally agree	11 (8.5)	7 (9.0)	4 (7.8)		
	Q28	Vigorous physical activity is contraindicated for this patient group	Strongly disagree	14 (10.8)	5 (6.3)	9 (17.6)	40.931	** < 0.001**
			Disagree	64 (49.2)	25 (31.6)	39 (76.5)		
			Agree	42 (32.3)	39 (49.4)	42 (5.9)		
			Totally agree	10 (7.7)	10 (12.7)	0 (0.0)		
**Factor 4—Physical functioning**	Q27	Physical activity increases joint function	Strongly disagree	1 (0.8)	0 (0.0)	1 (2.0)	6.969	0.073
			Disagree	7 (5.4)	7 (8.9)	0 (0.0)		
			Agree	81 (62.3)	50 (63.3)	31 (60.8)		
			Totally agree	41 (31.5)	22 (27.8)	19 (37.3)		
	Q29	I recommend physical activity	Strongly disagree	0 (0.0)	0 (0.0)	0 (0.0)	4.401	0.111
			Disagree	1 (0.8)	0 (0.0)	1 (2.0)		
			Agree	58 (45.0)	40 (51.3)	18 (35.3)		
			Totally agree	70 (54.3)	38 (48.7)	32 (62.7)		
	Q30	Balance training is important for this patient group	Strongly disagree	0 (0.0)	0 (0.0)	0 (0.0)	8.071	**0.018**
			Disagree	1 (0.8)	0 (0.0)	1 (2.0)		
			Agree	55 (42.6)	27 (34.2)	28 (56.0)		
			Totally agree	73 (56.6)	52 (65.8)	21 (42.0)		
	Q31	Maintaining normal body weight is important for this patient group	Strongly disagree	0 (0.0)	0 (0.0)	0 (0.0)	6.205	**0.013**
			Disagree	0 (0.0)	0 (0.0)	0 (0.0)		
			Agree	31 (24.2)	13 (16.7)	18 (36.0)		
			Totally agree	97 (75.8)	65 (83.3)	32 (64.0)		
**Factor 5—Knowledge**	Q32	I am familiar with WHO’s recommendation for moderate physical activity	Strongly disagree	10 (7.8)	7 (9.0)	3 (5.9)	5.516	0.138
			Disagree	26 (20.2)	11 (14.1)	15 (29.4)		
			Agree	65 (50.4)	44 (56.4)	21 (41.2)		
			Totally agree	28 (21.7)	16 (20.5)	12 (23.5)		
	Q33	The intensity (I.e. increased heart rate) of physical activity is important for this patient group	Strongly disagree	7 (0.8)	1 (1.3)	0 (0.0)	0.966	0.809
			Disagree	26 (20.2)	16 (20.5)	10 (19.6)		
			Agree	82 (63.6)	48 (61.5)	34 (66.7)		
			Totally agree	20 (15.5)	13 (16.7)	7 (13.7)		
	Q34	I am familiar with WHO’s recommendation for muscle strengthening exercise	Strongly disagree	16 (12.4)	8 (10.3)	8 (15.7)	2.882	0.410
			Disagree	36 (27.9)	19 (24.4)	17 (33.3)		
			Agree	67 (51.9)	45 (57.7)	22 (43.1)		
			Totally agree	10 (7.8)	6 (7.7)	4 (7.8)		

Statistically significant *p* value in bold

**Fig.1:**
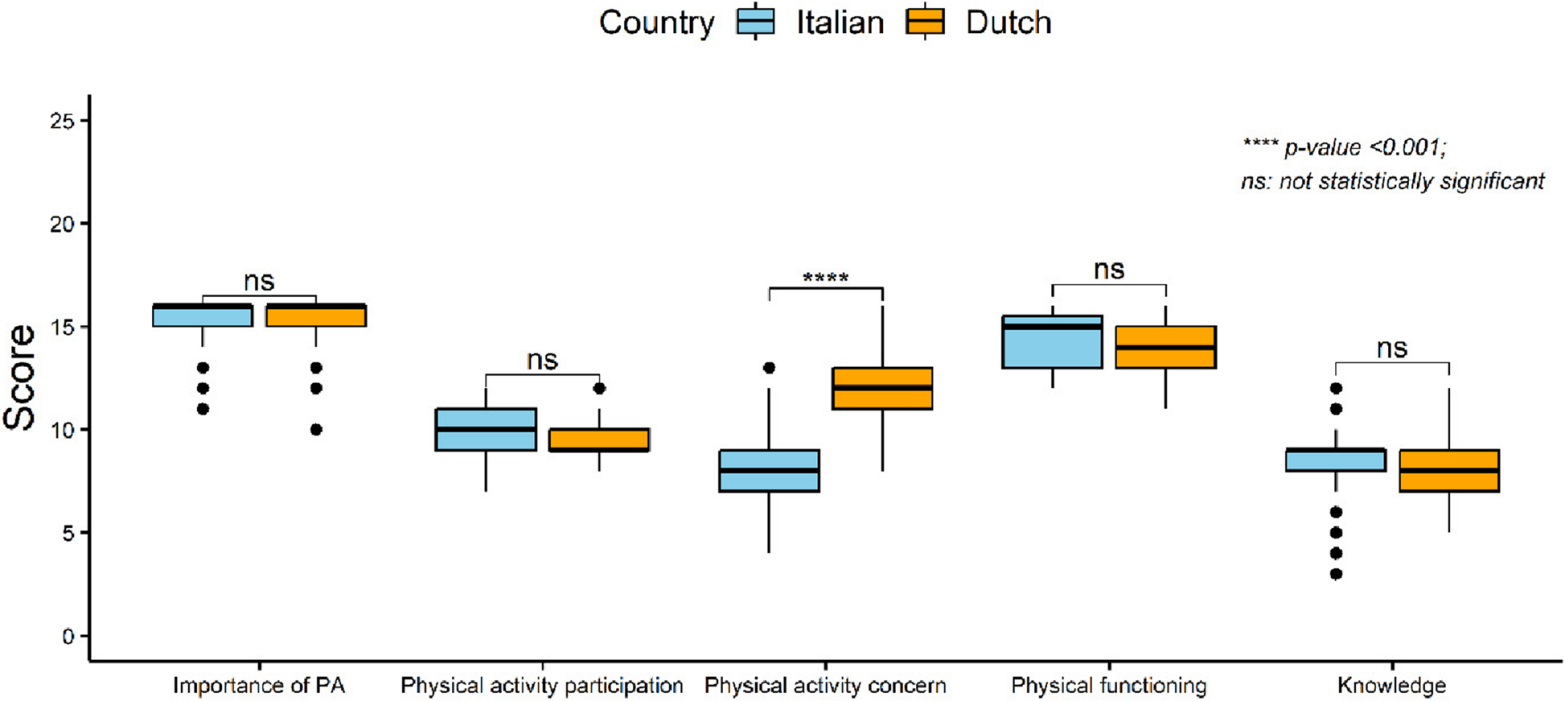
Comparison of sub-scores between Italian and Dutch surgeons

The sub-scores for the 5 identified factors were obtained by summing the scores of each item that belonged to that individual factor as determined by the factor analysis. The overall median score of orthopaedic surgeons’ attitude was 57 out of 72 (IQR: 54, 61), which is considered as “positive”. In particular, Dutch surgeons showed an 83.4% (60 out of 72) of the maximum total attitude score, while Italian ones showed 77.8% (56 out of 72). The comparison between countries showed that the total score was significantly higher in Dutch than Italian surgeons (*p* < 0.01). Comparing the sub-scores of factors 1, 2, 4, and 5 (“Importance of PA”, “Physical activity participation”, “Physical functioning”, and “Knowledge”) between both countries did not show significant differences (Fig. 1), while the sub-score for factor 3 “Physical activity concern” was significantly higher for Dutch surgeons than Italian ones (*p* < 0.001) (Table 4).

**Table 4:** Factors statistics

Factors	Total	Italy	The Netherlands	Mann–Whitney test
	Median (IQR)			Exact significance
Factor 1—Importance of PA	16 (15, 16)	16 (15,16)	16 (10,16)	0.491
Factor 2—Physical activity participation	9 (9, 11)	9 (9, 11)	9 (9, 10)	0.384
Factor 3—Physical activity concern	9 (8, 12)	8 (7, 9)	12 (11, 13)	** < 0.001**
Factor 4—Physical functioning	14 (13, 15)	15 (13, 15)	14 (13, 15)	0.602
Factor 5—Knowledge	9 (7, 9)	9 (8, 9)	8 (7, 9)	0.290
Total score	57 (54, 61)	56 (52, 58)	60 (55, 63)	** < 0.01**

Statistically significant *p* values in bold

### Factors influencing attitude towards physical activity

The results of the multiple linear regression analyses showed that, after accounting for other surgeon and clinic characteristics, the main variables significantly associated with the surgeons’ attitude were “Country” and “Type of clinic” (*P* < 0.05) (Table 5). This suggests that Dutch nationality was associated with a higher level of attitude, while working in more specialized structures (orthopedic clinics) was associated with a lower level of attitude. The model showed a significant overall fit, indicating that the independent variables collectively explained approximately 14.1% (F: 2.873; *p* < 0.05) of the variance in attitude.

**Table 5:** Regression analysis—Total score

	Unstandardized Coefficients	Standardized Coefficients		95.0% Confidence Interval for B	
	B	Beta	*P* value	Lower Bound	Upper Bound
(Constant)	43.907		** < 0.001**	32.365	55.449
Area	0.298	0.267	0.719	-1.343	1.940
Type of clinic	-1.646	0.038	**0.039**	-3.209	-0.083
Gender	0.920	-0.197	0.518	-1.896	3.736
Age	-0.495	0.061	0.597	-2.348	1.357
Education Level	0.602	-0.098	0.575	-1.524	2.729
Work experience in health service	1.781	0.057	0.182	-0.851	4.414
Work experience with this patient group	-0.882	0.360	0.500	-3.472	1.708
Sports or physical activity participation	1.312	-0.195	0.071	-0.115	2.738
Country	2.798	0.176	** < 0.018**	0.489	5.107
Adjusted R square	0.141				

Statistically significant *p* values in bold

Given the notable disparity observed in the “Physical activity concern” factor between the two countries, we additionally performed a multivariate linear regression analyses with its sub-score as dependent variable. Again, when adjusted for other surgeon and clinic characteristics, the variable “Country”, was significantly associated with the score on the “Physical activity concern” factor (*p* < 0.05) (Table 6). This indicates that Italian nationality was associated with a higher level of concern about PA among these individuals. This model also showed a significant overall fit, indicating that the independent variables accounted for approximately 59.0% (F: 18.578; *p* < 0.001) of the variance in “Physical activity concern”.

**Table 6:** Regression analysis—Factor 3 (“Physical activity concern”) Sub-score

	Unstandardized Coefficients	Standardized Coefficients		95.0% Confidence Interval for B	
	B	Beta	Sig	Lower Bound	Upper Bound
(Constant)	-1.639		0.446	-5.894	2.615
Area	-0.392	0.672	0.182	-0.972	0.187
Type of clinic	-0.095	-0.095	0.738	-0.658	0.468
Gender	0.655	-0.021	0.215	-0.386	1.696
Age	-0.042	0.078	0.902	-0.711	0.628
Education Level	0.685	-0.015	0.079	-0.081	1.451
Work experience in health service	0.317	0.120	0.517	-0.649	1.282
Work experience with this patient group	-0.107	0.119	0.825	-1.063	0.849
Sports or physical activity participation	-0.087	-0.044	0.740	-0.604	0.430
Country	3.779	-0.022	** < 0.001**	2.952	4.606
Adjusted R square	0.590				

Statistically significant *p* values in bold

## Discussion

To the best of our knowledge, this is the first study investigating orthopaedic surgeons’ attitude towards PA for people after THR and TKR, as well as cross-cultural differences between a Southern European country, represented by Italy, and a Northern European country, represented by the Netherlands.

### Attitude towards physical activity

Overall orthopaedic surgeons’ attitude towards PA for people after THR and TKR was “positive” (79.2%; median score of 57 out of 72). Specifically, only 15.7% obtained a "neutral" score, with no surgeons reporting a "negative" score. This is in line with the study of Gnanendran et al. [19], who found that 95% of clinicians have a positive attitude towards exercise counseling.

### Comparison between a Northern and Southern European country

The comparison of the total attitude score between Italian and Dutch surgeons showed that Dutch surgeons were more positive towards PA for people after THR and TKR compared to the Italian (Table 4). Specifically, a difference was found in the “Physical activity concern” factor, where Dutch orthopaedic surgeons showed to be more liberal and were less concerned about negative effects of PA on the survival of the prostheses, whereas Italian orthopaedic surgeons showed more concern. It can be suggested that the aforementioned results are in line with the observation that the general Dutch population is more into sport, exercise, and PA participation compared to the Italian [22]. On the contrary, factors “Importance of PA”, “Physical activity participation”, “Physical functioning”, and “Knowledge” did not show differences between countries.

Surprisingly, the attitude towards PA did not seem to be influenced by the own PA and sports participation of the surgeon. In contrast with other authors [23, 24], the correlation in our study between own sport participation and the attitude towards PA was not statistically significant (*p* = 0.071). Fie et al. [23] found that a higher personal PA level of physicians and nurses is associated with higher physical activity-promoting practices. Moreover, Thaler et al. [24] found that orthopaedic surgeons with a higher level of PA were more inclined to recommend earlier return to sport activities for patients undergoing THR. Based on the literature we did not have a clear explanation for these findings.

The attitude among surgeons differed significantly based on country of residence and clinical setting in which they work. These findings question if cultural, socioeconomic, and/or contextual factors associated with different countries influence surgeons’ attitude. In Italy, the total number of orthopaedic surgeons in 2019 was 9,085 [27] equivalent to 1.52 per 10,000 inhabitants. Furthermore, the healthcare system is primarily funded through general taxation and social security contribution, offering public and free-of-charge services to all citizens. The Netherlands had a total of 876 orthopaedic surgeons, equivalent to 0.51 per 10,000 in 2018 [28, 29]. Health insurance, provided by private companies, is required for all citizens of the Netherlands. Most patients in the Netherlands undergo fast-track surgery and are discharged from the hospital within 3 days. Although there is no standardized rehabilitation program upon discharge, it is recommended that patients follow physiotherapy [30]. The surgery and rehabilitation procedures in both the Netherlands and Italy are quite similar. These are just a few examples of the many differences ans imilarities between countries, highlighting that the results within each country should be interpreted within the specific cultural, social, and economic context of each respective country.

### Factors influencing attitude towards physical activity

To investigate our data more in depth, we performed a multiple linear regression analysis to explore the variables that might influence the overall attitude towards PA, as well as the “Physical activity concern” factor as a separate one. This factor is particularly important since it may potentially compromise individual’s health and overall wellbeing and it may have an adverse impact on clinician’s counselling practices [19]. As in the analysis of variables influencing the overall attitude, country of residence exhibited a substantial and significant effect. In contrast to the analysis of the overall attitude, the type of clinic was not found to influence the attitude towards PA. This suggests that the work environment may not be relevant to influence the level of concern about the impact of PA on patients after THR and TKR.

After the first post-operative rehabilitation period, engaging in PA and sport activities have been demonstrated to improve the general health and fitness, relieving pain and joint stiffness, enhancing physical function, and minimizing the risk of falls [31–33]. Indeed, for patients who have undergone THR and TKR, PA and sport activities remain crucial to enhance fitness, health status, and social contact [24]. In particular, the improvement in persons’ fitness can contribute to greater independence in daily life activities. However, such activities should be approved, and advised by orthopaedic surgeons. Indeed, people after THR and TKR should rely on surgeon's approval and advise to perform physical and sport activities. Therefore, it is crucial that the orthopaedic surgeon, the figure people trust and should refer to, invests time to encourage and inform, as much as possible, patients to be more active and to perform PA within the bound of the allowed and suggested activities [19, 34]. However, there is a mismatch between the patients’ needs and the counseling provided by the doctor [19, 34]. Gnanendran et al. [19] found that 32% of clinicians never or rarely discuss PA with patients, while 60% of patients reported never or rarely receiving counseling from their doctor.

In general, individuals who have undergone total knee or hip replacement surgery (and completed the subsequent rehabilitation period) should be considered “healthy” again. Therefore, they should be advised to adhere to the general recommendations provided by the WHO about PA [14]. At the same time, it is important for those people to avoid activities that are not recommended while following international consensus [4]. Most surgeons agree on the activities that should be allowed, such as low-impact sports activities (walking, swimming, and biking on level surfaces) [4, 16, 24], and not allowed, such as contact sports, most of the ball sports, and martial arts [4, 12, 24]. However, such studies did not investigate differences in attitude towards PA of surgeons between countries. A possible strategy to improve the attitude towards PA and, consequently, exercise counseling could involve effective health promotion and disease prevention among medical students [35]. Furthermore, future studies are needed to explore modifiable factors and strategies that could positively influence orthopaedic surgeons’ counseling on PA for people after THR and TKR.

This study presents some limitations. The differences observed among surgeons in terms of sample characteristics could be due to selection bias during the recruitment process. In particular, in The Netherlands, the distribution of the questionnaire was initiated in a University Hospitalusing the snowball approach. Also, there was a difference in the sample size of the two groups. However, this difference reflects the different proportion of surgeons per inhabitants in Italy and the Netherlands. Third, factors beyond the scope of this study, such as personal childhood experiences, which could potentially influence surgeons’ attitude towards PA, were not examined. Finally, it is possible that surgeons overestimated their attitude (social-desirability bias [35]) while responding to the questionnaire.

## Conclusion

Since the renowned positive impact of PA and sports on people’s fitness and general health, it becomes crucial for orthopaedic surgeons to advise an active lifestyle for people after TKR and THR. Overall orthopaedic surgeons’ attitude towards PA was positive (79.2%, 57 out of 72). Specifically, Dutch surgeons showed a more positive attitude compared to Italian ones. This highlights the need for further research to untangle specific factors, such as cultural, socioeconomic, or contextual differences within the variable “country” that may influence orthopaedic surgeons’ attitudes towards PA.

### Supplementary Information

Supplementary Material 1.

## Data Availability

The datasets used and/or analysed during the current study available from the corresponding author on reasonable request.

## Abbreviations

OA: Osteoarthritis
PA: Physical activity
THR: Total hip replacement
TKR: Total knee replacement
OECD: Organization for Economic Co-operation and Development
IOR: Rizzoli Orthopaedic Institute
NTNU: Norwegian University of Science and Technology
UMCG: University Medical Center Groningen
EFA: Exploratory factor analysis
