# Supplementary material for: Orthopaedic surgeons' attitude toward physical activity for people after total hip or knee replacement: Northern vs Southern European country
Source: BMC Musculoskelet Disord. 2024 May 11;25:371. doi: 10.1186/s12891-024-07488-w (PMC11088017; doi:10.1186/s12891-024-07488-w)
Supplement: Supplementary file 1 — Supplementary Material 1. [file 12891_2024_7488_MOESM1_ESM.docx]

Additional file

Table S1. STROBE Statement—Checklist of items that should be included in reports of cross-sectional studies

|  | Item No | Recommendation | Page No |
| --- | --- | --- | --- |
| **Title and abstract** | 1 | (*a*) Indicate the study’s design with a commonly used term in the title or the abstract | 1 |
|  |  | (*b*) Provide in the abstract an informative and balanced summary of what was done and what was found | 2-3 |
| Introduction | | |  |
| Background/rationale | 2 | Explain the scientific background and rationale for the investigation being reported | 4 |
| Objectives | 3 | State specific objectives, including any prespecified hypotheses | 5 |
| Methods | | |  |
| Study design | 4 | Present key elements of study design early in the paper | 5 |
| Setting | 5 | Describe the setting, locations, and relevant dates, including periods of recruitment, exposure, follow-up, and data collection | 6 |
| Participants | 6 | (*a*) Give the eligibility criteria, and the sources and methods of selection of participants | 7 |
| Variables | 7 | Clearly define all outcomes, exposures, predictors, potential confounders, and effect modifiers. Give diagnostic criteria, if applicable | 6 |
| Data sources/ measurement | 8* | For each variable of interest, give sources of data and details of methods of assessment (measurement). Describe comparability of assessment methods if there is more than one group | *6* |
| Bias | 9 | Describe any efforts to address potential sources of bias | 7 |
| Study size | 10 | Explain how the study size was arrived at | 9 |
| Quantitative variables | 11 | Explain how quantitative variables were handled in the analyses. If applicable, describe which groupings were chosen and why | 8 |
| Statistical methods | 12 | (*a*) Describe all statistical methods, including those used to control for confounding | 8 |
|  |  | (*b*) Describe any methods used to examine subgroups and interactions | 8 |
|  |  | (*c*) Explain how missing data were addressed | 9 |
|  |  | (*d*) If applicable, describe analytical methods taking account of sampling strategy |  |
|  |  | (*e*) Describe any sensitivity analyses | 8-9 |
| Results | | |  |
| Participants | 13* | (a) Report numbers of individuals at each stage of study—eg numbers potentially eligible, examined for eligibility, confirmed eligible, included in the study, completing follow-up, and analysed | 9 |
|  |  | (b) Give reasons for non-participation at each stage | - |
|  |  | © Consider use of a flow diagram | - |
| Descriptive data | 14* | (a) Give characteristics of study participants (eg demographic, clinical, social) and information on exposures and potential confounders | 9 |
|  |  | (b) Indicate number of participants with missing data for each variable of interest | 9-14 |
| Outcome data | 15* | Report numbers of outcome events or summary measures | 11 |
| Main results | 16 | (*a*) Give unadjusted estimates and, if applicable, confounder-adjusted estimates and their precision (eg, 95% confidence interval). Make clear which confounders were adjusted for and why they were included | 11 |
|  |  | (*b*) Report category boundaries when continuous variables were categorized | 12 |
|  |  | © If relevant, consider translating estimates of relative risk into absolute risk for a meaningful time period |  |
| Other analyses | 17 | Report other analyses done—eg analyses of subgroups and interactions, and sensitivity analyses | 12-17 |
| Discussion | | |  |
| Key results | 18 | Summarise key results with reference to study objectives | 17 |
| Limitations | 19 | Discuss limitations of the study, taking into account sources of potential bias or imprecision. Discuss both direction and magnitude of any potential bias | 20 |
| Interpretation | 20 | Give a cautious overall interpretation of results considering objectives, limitations, multiplicity of analyses, results from similar studies, and other relevant evidence | 18 |
| Generalisability | 21 | Discuss the generalisability (external validity) of the study results | 20 |
| Other information | | |  |
| Funding | 22 | Give the source of funding and the role of the funders for the present study and, if applicable, for the original study on which the present article is based | 22 |

*Give information separately for exposed and unexposed groups.

**Note:** An Explanation and Elaboration article discusses each checklist item and gives methodological background and published examples of transparent reporting. The STROBE checklist is best used in conjunction with this article (freely available on the Web sites of PLoS Medicine at http://www.plosmedicine.org/, Annals of Internal Medicine at http://www.annals.org/, and Epidemiology at http://www.epidem.com/). Information on the STROBE Initiative is available at www.strobe-statement.org.

Table S2. Questionnaire investigating attitude towards physical activity for people after THR and TKR (section 1 - Background setting)

| **Background setting** | | | | | |
| --- | --- | --- | --- | --- | --- |
| Questions | | Answers | | | |
| Q1 | State |  | | | |
| Q2 | Area | Rural | Suburban | Urban |  |
| Q3 | Type of clinic | Orthopaedic  clinic | Hospital  policlinic | Community  health clinic | Physiotherapy clinic |
|  |  | Health  Studio | Rehabilitation  clinic |  |  |
| Q4 | Profession | General  Physician | Rehabilitation  Physician | Orthopaedic  surgeon | Physiotherapist |
|  |  | Nurse | Occupational  therapist | Medical  student | Exercise trainer  (non-medical) |
| Q5 | Gender | Male | Female | Other |  |
| Q6 | Age (years) | < 30 | 31-40 | 41-50 | 51-60 |
|  |  | 61-70 | > 70 |  |  |
| Q7 | Educational level | Diploma | Bachelor  degree | Master  degree | PhD degree |
| Q8 | Work experience in health service (years) | < 1 | 1-5 | 6-10 | 11-20 |
|  |  | > 20 |  |  |  |
| Q9 | Work experience with this patient group (years) | < 1 | 1-5 | 6-10 | 11-20 |
|  |  | > 20 |  |  |  |
| Q10 | Sports or physical activity participation | None | Leisure /  irregular | Moderate /  regular | High /  competitive |

Table S3. Questionnaire investigating attitude towards physical activity for people after THR and TKR (section 2 - Background setting)

| **Personal information** | | | | | |
| --- | --- | --- | --- | --- | --- |
| Questions | | Answers | | | |
| Q5 | Gender | Male | Female | Other |  |
| Q6 | Age (years) | < 30 | 31-40 | 41-50 | 51-60 |
|  |  | 61-70 | > 70 |  |  |
| Q7 | Educational level | Diploma | Bachelor  degree | Master  degree | PhD degree |
| Q8 | Work experience in health service (years) | < 1 | 1-5 | 6-10 | 11-20 |
|  |  | > 20 |  |  |  |
| Q9 | Work experience with this patient group (years) | < 1 | 1-5 | 6-10 | 11-20 |
|  |  | > 20 |  |  |  |
| Q10 | Sports or physical activity participation | None | Leisure /  irregular | Moderate /  regular | High /  competitive |

Table S4. Questionnaire investigating attitude towards physical activity for people after THR and TKR (section 3 – Personal information)

| **Clinic information** | | | | | |
| --- | --- | --- | --- | --- | --- |
| Questions | | Answers | | | |
| Q11 | The clinic offers exercise classes for this patient group | None | Daily | Weekly | Occasional |
| Q12 | The clinic offers a pre-operative exercise program | None | < 3 times introductory | Weekly 1-2 months | Weekly > 2 months |
| Q13 | The clinic offers a post-operative exercise program | None | < 3 times introductory | Weekly 1-2 months | Weekly > 2 months |
| Q14 | Advice and supervision are individually personalized | No | Yes |  |  |
| Q15 | The information is given | None | Orally | Written | Orally and  written |
| Q16 | I give physical activity advice | Not my job | Never | Sometimes | Always |
| Q17 | I give smoke secession advice | Not my job | Never | Sometimes | Always |
| Q18 | I give weight reduction advice | Not my job | Never | Sometimes | Always |
| Q19 | The clinic gives information about importance of physical activity by the | None | Physician | Secretary | Nurse |
|  |  | Physiotherapist | Exercise trainer (non-medical) | Occupational therapist |  |

Table S5. Questionnaire investigating attitude towards physical activity for people after THR and TKR (section 4 – Attitude towards physical activity)

| **Attitudes towards physical activity** | | | | | |
| --- | --- | --- | --- | --- | --- |
| **Questions** | | **Answers** | | | |
|  |  | Strongly disagree | Disagree | Agree | Totally agree |
| Q20 | Physical activity is important for general health | 1 | 2 | 3 | 4 |
| Q21 | Physical activity is important for physical function | 1 | 2 | 3 | 4 |
| Q22 | Physical activity is important for quality of life | 1 | 2 | 3 | 4 |
| Q23 | Physical activity is for everyone | 1 | 2 | 3 | 4 |
| Q24 | The prosthesis alone restores full physical function | 4 | 3 | 2 | 1 |
| Q25 | Physical activity is not necessary | 4 | 3 | 2 | 1 |
| Q26 | Vigorous physical activity may damage the prosthesis | 4 | 3 | 2 | 1 |
| Q27 | Physical activity increases joint function | 1 | 2 | 3 | 4 |
| Q28 | Vigorous physical activity is contraindicated for this patient group | 4 | 3 | 2 | 1 |
| Q29 | I recommend physical activity | 1 | 2 | 3 | 4 |
| Q30 | Balance training is important for this patient group | 1 | 2 | 3 | 4 |
| Q31 | Maintaining normal body weight is important for this patient group | 1 | 2 | 3 | 4 |
| Q32 | I am familiar with WHO’s recommendation for moderate physical activity | 1 | 2 | 3 | 4 |
| Q33 | The intensity (I.e. increased heart rate) of physical activity is important for this patient group | 1 | 2 | 3 | 4 |
| Q34 | I am familiar with WHO’s recommendation for muscle strengthening exercise | 1 | 2 | 3 | 4 |
| Q35 | Exercises for muscle strength is important for function for this patient group | 1 | 2 | 3 | 4 |
| Q36 | Physical activity is important to enable participation (social, work, leisure) | 1 | 2 | 3 | 4 |
| Q37 | Physical activity is important for coping with having prosthesis | 1 | 2 | 3 | 4 |

Table S6. Total variance explained

| Component | Initial Eigenvalues | | | Extraction Sums of Squared Loadings | | | Rotation Sums of Squared Loadings | | |
| --- | --- | --- | --- | --- | --- | --- | --- | --- | --- |
|  | Total | % of Variance | Cumulative % | Total | % of Variance | Cumulative % | Total | % of Variance | Cumulative % |
| 1 | 4.633 | 25.741 | 25.741 | 4.633 | 25.741 | 25.741 | 2.777 | 15.429 | 15.429 |
| 2 | 2.344 | 13.020 | 38.761 | 2.344 | 13.020 | 38.761 | 2.431 | 13.504 | 28.932 |
| 3 | 1.920 | 10.669 | 49.431 | 1.920 | 10.669 | 49.431 | 2.349 | 13.052 | 41.984 |
| 4 | 1.465 | 8.140 | 57.571 | 1.465 | 8.140 | 57.571 | 2.150 | 11.943 | 53.927 |
| 5 | 1.235 | 6.861 | 64.432 | 1.235 | 6.861 | 64.432 | 1.891 | 10.504 | 64.432 |
| 6 | 0.959 | 5.328 | 69.760 |  |  |  |  |  |  |
| 7 | 0.803 | 4.460 | 74.220 |  |  |  |  |  |  |
| 8 | 0.721 | 4.004 | 78.224 |  |  |  |  |  |  |
| 9 | 0.616 | 3.422 | 81.646 |  |  |  |  |  |  |
| 10 | 0.593 | 3.292 | 84.938 |  |  |  |  |  |  |
| 11 | 0.542 | 3.011 | 87.949 |  |  |  |  |  |  |
| 12 | 0.464 | 2.577 | 90.527 |  |  |  |  |  |  |
| 13 | 0.381 | 2.119 | 92.645 |  |  |  |  |  |  |
| 14 | 0.350 | 1.946 | 94.591 |  |  |  |  |  |  |
| 15 | 0.305 | 1.694 | 96.285 |  |  |  |  |  |  |
| 16 | 0.281 | 1.558 | 97.843 |  |  |  |  |  |  |
| 17 | 0.254 | 1.412 | 99.255 |  |  |  |  |  |  |
| 18 | 0.134 | 0.745 | 100.000 |  |  |  |  |  |  |
| *Extraction Method: Principal Component Analysis.* | | | | | | | | | |

Table S7. Exploratory factor analysis – Rotated component matrix

|  | | | | | |
| --- | --- | --- | --- | --- | --- |
|  | Component | | | | |
|  | 1 | 2 | 3 | 4 | 5 |
| Physical activity is important for general health | 0.900 |  |  |  |  |
| Physical activity is important for physical function | 0.842 |  |  |  |  |
| Physical activity is important for quality of life | 0.812 |  |  |  |  |
| Physical activity is for everyone | 0.568 | 0.322 |  |  |  |
| Physical activity is important to enable participation (social, work, leisure) |  | 0.752 |  |  |  |
| Exercises for muscle strength is important for function for this patient group |  | 0.741 |  |  |  |
| Physical activity is important for coping with having prosthesis |  | 0.623 |  | 0.350 |  |
| Physical activity is not necessary |  |  | 0.794 |  |  |
| The prosthesis alone restores full physical function |  |  | 0.775 |  |  |
| Vigorous physical activity is contra-indicated for this patient group |  | -0.328 | 0.720 |  |  |
| Vigorous physical activity may damage the prosthesis |  | -0.365 | 0.526 |  |  |
| Physical activity increases joint function |  |  |  | 0.751 |  |
| I recommend physical activity |  |  |  | 0.691 |  |
| Balance training is important for this patient group |  |  |  | 0.633 |  |
| Maintaining normal body weight is important for this patient group |  |  | 0.336 | 0.535 |  |
| I am familiar with WHOs recommendation for muscle strengthening exercise |  |  |  |  | 0.876 |
| I am familiar with WHOs recommendation for moderate physical activity |  |  |  |  | 0.822 |
| The intensity (I.e. increased heart rate) of physical activity is important for this patient group |  | 0.488 |  |  | 0.528 |
| *Extraction Method: Principal Component Analysis.*  *Rotation Method: Varimax with Kaiser Normalization.^a^* | | | | | |
| *a. Rotation converged in 5 iterations.* | | | | | |
